# High-sensitivity troponin T as a predictor of cardiovascular events in colorectal cancer patients undergoing chemotherapy: results of the COLOVASC prospective cohort study

**DOI:** 10.3389/fcvm.2026.1943142

**Published:** 2026-10-13

**Authors:** Bence Prohászka, Zsófia Sándor, Tamás Ferenci, László Gráf, Júlia Lohinszky, Anna Horváth, Bálint Tegze, Katalin Éva Tóth, Tamás Masszi, Zsófia Dóra Drobni, Daniela Maria Cardinale, Zoltán Pozsonyi

**Affiliations:** 1 Department of Internal Medicine and Hematology, Semmelweis University, Budapest, Hungary; 2 Department of Cardiology, Heart and Vascular Center, Semmelweis University, Budapest, Hungary; 3 Physiological Controls Research Center, Obuda University, Budapest, Hungary; 4 Department of Statistics, Corvinus University of Budapest, Budapest, Hungary; 5 Cardioncology Unit, Cardioncology and Second Opinion Division, European Institute of Oncology IRCCS, Milan, Italy

**Keywords:** cardio-oncology, cardiovascular toxicity, chemotherapy, colorectal cancer, major cardiovascular events, risk stratification, troponin

## Abstract

**Background:**

Fluoropyrimidine-based chemotherapy is associated with cardiovascular events in patients with colorectal cancer, yet validated tools for pre-treatment cardiovascular risk stratification are lacking. Cardiac biomarkers are not routinely incorporated into baseline assessment and prospective evidence supporting their use remains limited.

**Objectives:**

Our aim was to determine whether baseline high-sensitivity troponin T (hs-TnT) provides prognostic information for major adverse cardiovascular events during chemotherapy in patients with colorectal cancer.

**Materials and methods:**

Within a prospective single-center observational cohort study at the Department of Internal Medicine and Hematology, Semmelweis University, consecutive patients with histologically confirmed newly diagnosed or recurrent colorectal cancer scheduled to receive 5-fluorouracil (5-FU)-based chemotherapy were enrolled between September 1, 2020, and June 2, 2024. Baseline hs-TnT concentrations were measured before treatment initiation, and patients were followed for the occurrence of major adverse cardiovascular events (MACEs). Independent predictors of MACE were identified using penalized multivariable logistic regression. Receiver operating characteristic (ROC) curve analysis evaluated the discriminatory performance of baseline hs-TnT and determined the optimal cut-off value using the Youden index. Adequacy index analysis assessed the relative prognostic contribution of hs-TnT to the multivariable model.

**Results:**

180 patients were enrolled, with a median follow-up of 185 days, 44 patients (24.4%) experienced MACE, more than half of them occurred within the first two cycles of chemotherapy. Baseline hs-TnT demonstrated modest discrimination for predicting MACE (AUC 0.628, 95% CI 0.513–0.742). The optimal hs-TnT cut-off was 14 ng/L. In a multivariable analysis, elevated baseline hs-TnT (OR 2.35 per +10 ng/L, 95% CI 1.37–4.05) and Vascular Endothelial Growth Factor (VEGF)-inhibitor therapy (OR 2.38, 95% CI 1.23–4.60) were associated with MACEs. In a sensitivity analysis, excluding treatment-related hypertension from the composite endpoints, elevated baseline hs-TnT remained the only independent predictor. According to the adequacy index analysis, hs-TnT contributed 38.3% of the total prognostic information provided by the multivariable model.

**Conclusions:**

Baseline hs-TnT was independently associated with MACEs in patients with colorectal cancer receiving fluoropyrimidine-based chemotherapy. Although its discriminatory performance as a standalone biomarker was modest, hs-TnT provided incremental prognostic information for baseline cardiovascular risk assessment and may help identify patients who warrant closer cardiovascular surveillance.

## 1 Introduction

Colorectal cancer (CRC) is the third most common malignancy and the second leading cause of cancer-related mortality worldwide (1, 2), and fluoropyrimidine-based chemotherapy — mainly 5-fluorouracil (5-FU) and its derivatives — remains a cornerstone of systemic treatment (3). While these therapies improve survival, they may precipitate clinically relevant cardiovascular (CV) toxicity, including ischemic events, arrhythmias, and treatment-associated hypertension (4–10). This risk may be further amplified in patients receiving vascular endothelial growth factor (VEGF)-inhibitor therapy, which is widely used in metastatic CRC and is mechanistically linked to endothelial dysfunction, blood-pressure elevation, and vascular complications (11, 12). Identifying patients at increased CV risk before chemotherapy is therefore a critical step to enable targeted monitoring and preventive care.

Recent cardio-oncology guidelines emphasize pre-treatment CV risk stratification (13); however, the evidence base supporting current approaches is heterogeneous and largely derived from other cancer populations, such as anthracycline-, HER2-, or immune-checkpoint-inhibitor–treated cohorts (13). In contrast, no validated, biomarker-based risk-stratification strategy exists for CRC patients receiving 5-FU and/or VEGF-inhibitor therapy, and cardiac biomarkers are not routinely incorporated into clinical decision-making in this setting. As a result, surveillance intensity is typically uniform rather than risk-directed, potentially leading to both under-monitoring of vulnerable patients and unnecessary evaluations in low-risk individuals (14, 15).

High-Sensitivity Troponin T (hs-TnT) is a sensitive marker of subclinical myocardial injury and has been associated with adverse CV outcomes in several oncologic contexts (16–18); however, its prognostic role at baseline — prior to chemotherapy initiation — has not been prospectively evaluated in CRC populations (19, 20). Whether hs-TnT can provide clinically meaningful, implementable information to triage patients toward closer early-phase CV follow-up, and how this relates to treatment complexity such as VEGF-inhibitor exposure, remains unknown.

Therefore, we conducted a prospective cohort study of patients with CRC receiving fluoropyrimidine-based chemotherapy to determine whether baseline hs-TnT independently predicts MACEs, identify additional prognostic determinants, including VEGF-inhibitor therapy, and assess the incremental prognostic value of hs-TnT beyond conventional clinical characteristics. Our overarching aim was to inform biomarker-guided cardiovascular risk stratification in routine colorectal cancer care.

## 2 Materials and methods

### 2.1 Ethical approval

The study was approved by the Scientific and Research Ethics Committee of the Medical Research Council (ETT TUKEB), Hungary, under the unique identifier IV/617-1/2021/EKU. Further information is available at https://ett.okfo.gov.hu/eng2021/.

### 2.2 Study design and population

We conducted a prospective, single-center observational cohort study at the Department of Internal Medicine and Hematology, Semmelweis University, between September 1, 2020, and June 2, 2024. The study is reported in accordance with the Strengthening the Reporting of Observational Studies in Epidemiology (STROBE) statement. Consecutive adult patients with histologically confirmed colorectal cancer (CRC) who were scheduled to receive fluoropyrimidine-based chemotherapy, with or without VEGF-inhibitor therapy, were prospectively enrolled prior to treatment initiation.

The planned study period was consequently prolonged due to COVID-19-related hospital restrictions, which substantially limited patient enrollment. Patients were enrolled prior to treatment initiation following multidisciplinary tumor-board approval. The primary inclusion criteria for the study included a histologically confirmed diagnosis of CRC, either newly diagnosed or recurrent. For recurrent CRC, a therapy-free period of at least six months was required. Only patients scheduled for chemotherapy were included in the study. Key exclusion criteria were concurrent active malignancy, ongoing chemotherapy, and inability to ensure longitudinal follow-up. Prior to enrollment, all patients received standardized verbal and written information about the study and subsequently provided written informed consent.

Of 1,666 patients evaluated by the tumor board during the enrollment period, 222 met the protocol-defined eligibility criteria; 40 treated in external institutions were excluded due to uncertain follow-up and two declined participation, yielding an analytic cohort of 180 patients (Figure 1). No formal sample size calculation was performed because the study was designed as an exploratory prospective observational cohort. Consecutive eligible patients presenting during the study period were enrolled. To ensure reliable follow-up and complete outcome capture, follow-up was aligned with the standard adjuvant chemotherapy schedule (≈24 weeks) with a planned minimum duration of six months, extended in metastatic disease while maintenance therapy continued; patients receiving neoadjuvant therapy were followed from the first dose, and follow-up was discontinued two weeks after treatment cessation. Complete follow-up was achieved for all enrolled participants. Potential sources of bias were minimized through prospective consecutive enrollment, predefined eligibility criteria, standardized baseline data collection, complete follow-up, and prespecified statistical analyses.

**Figure 1:**
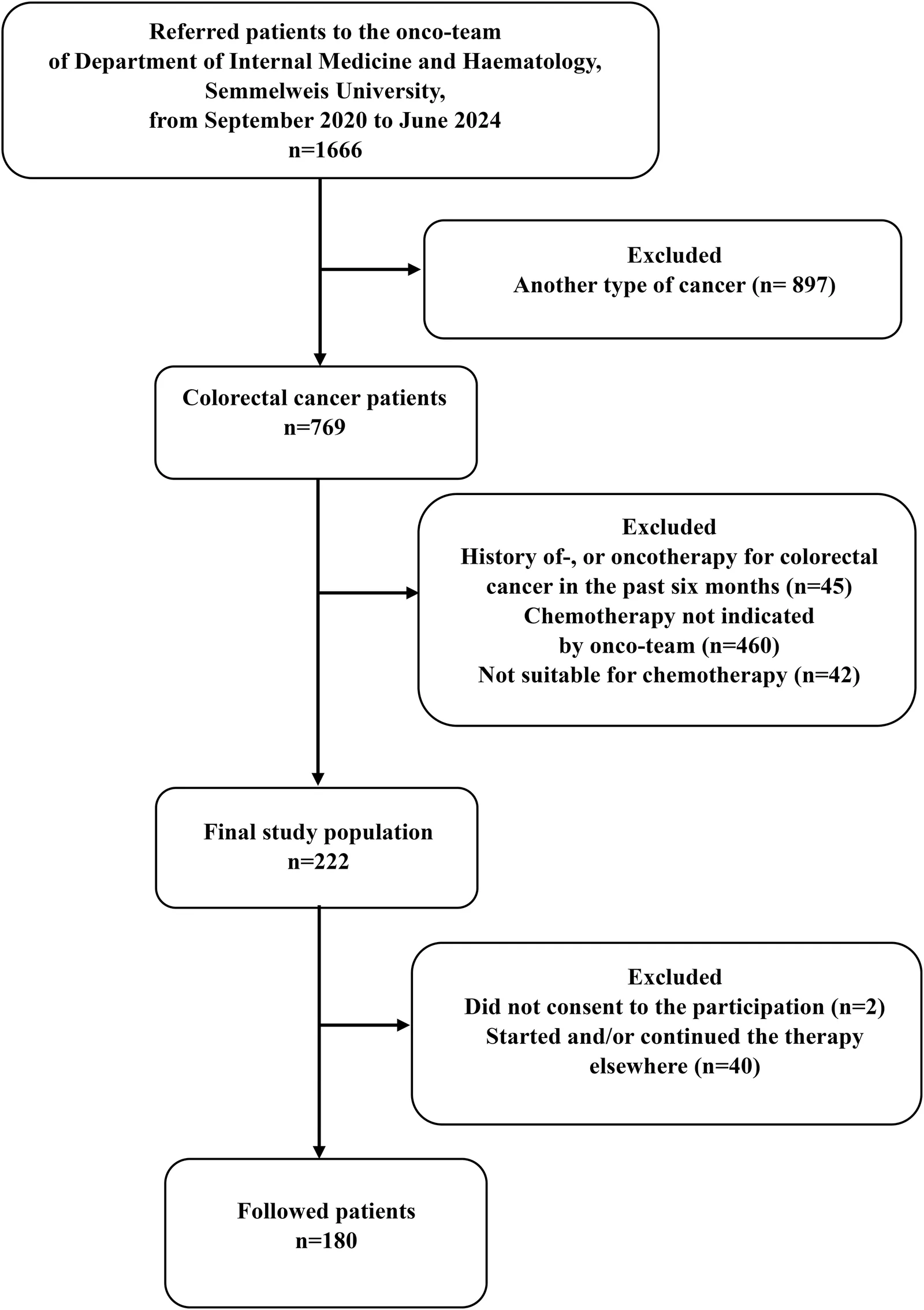
Patient selection flowchart.

Baseline demographics, comorbidities, CV history, medications, cancer characteristics (metastatic vs. non-metastatic disease, treatment intent), and planned systemic therapy were recorded prior to the first chemotherapy cycle using a structured questionnaire and the electronic hospital information system. Pre-existing cardiovascular diseases were not considered exclusion criteria, as the study aimed to evaluate cardiovascular risk in a real-world population of patients with colorectal cancer undergoing chemotherapy. Information on clinical conditions potentially associated with elevated hs-TnT levels, including prior myocardial infarction, heart failure, hypertension, stroke/transient ischemic attack, and impaired renal function, was collected at baseline and considered in the subsequent analyses. The Charlson Comorbidity Index (CCI) was calculated as a summary measure of pre-existing comorbidity, and VEGF-inhibitor therapy was prespecified as a marker of treatment complexity and vascular-risk exposure (21).

Right-sided CRC was defined as tumors extending from the ileocecal valve to the left colic flexure.

### 2.3 Biomarker assessment

Baseline hs-TnT was measured prior to chemotherapy initiation in the central laboratory using a standardized Roche high-sensitivity immunoassay, and blood samples were also obtained before each chemotherapy cycle as part of routine monitoring. The assay is specifically validated for quantitative determination of hs-TnT in human serum and plasma. In accordance with established reference limits, the 99th percentile upper reference limit (URL) in the general population is 14 ng/L (pg/mL) (95% CI: 12.7–24.9 ng/L), with sex-specific values of 9.0 ng/L in females and 16.8 ng/L in males. Analytical performance characteristics were as follows: limit of blank 2.5 ng/L, limit of detection 3 ng/L, and functional sensitivity (coefficient of variation ≤10%) 13 ng/L. Values >14 ng/L were considered above the upper reference limit and triggered clinical notification of the treating oncologist; cardiology consultation, including echocardiography when clinically indicated, was arranged in these cases. Although serial on treatment hs-TnT values were recorded throughout chemotherapy, the present prognostic analysis focused on baseline hs-TnT as a pre-treatment risk marker, and serial measurements were not incorporated into the predictive models.

### 2.4 Follow-up and outcome definition

Patients were followed prospectively, and medical records were reviewed after each chemotherapy cycle to identify incident CV events. The primary outcome was the occurrence of MACE during follow-up, defined *a priori* as any of the following new diagnoses or clinical events: treatment-associated hypertension, atrial fibrillation or flutter, angina or ischemic heart disease, acute coronary syndrome, heart failure, stroke or transient ischemic attack, and venous thromboembolic events (deep vein thrombosis, pulmonary embolism, or portal vein thrombosis) (22, 23). Event definitions adhered to standardized clinical criteria and were verified from physician documentation and diagnostic test reports.

### 2.5 Statistics

There were no missing data for any variables included in the analyses; therefore, no imputation was required. Continuous variables were retained on their original scale whenever possible to preserve statistical information and avoid unnecessary categorization. Data are presented as medians with interquartile ranges for continuous variables and as counts with percentages for categorical variables. To identify clinical factors potentially associated with elevated baseline hs-TnT levels, including pre-existing cardiovascular conditions and other relevant comorbidities, univariable analyses were performed using the Mann–Whitney U test (Wilcoxon rank-sum test) for continuous variables and the Pearson's chi-square test for categorical variables. Subsequently, variables of clinical relevance were entered into a multivariable logistic regression model. Variables evaluated included estimated glomerular filtration rate (eGFR), age, prior acute myocardial infarction, hypertension, stroke/TIA, heart failure, and Charlson Comorbidity Index values >6.

The primary analysis modeled the occurrence of MACE as a binary outcome, reflecting its relevance for early-phase clinical decision-making during chemotherapy and the short, protocol-driven follow-up window; although event times were recorded, the study was not powered for formal time-to-event modeling.

To limit model overfitting in the context of a modest event count, prespecified predictors were evaluated using an L2-penalized multivariable logistic regression model, with the penalty parameter selected according to the corrected Akaike Information Criterion (24). The primary multivariable model for MACE included the following prespecified covariates, selected on the basis of clinical relevance and established cardiovascular risk factors, and blinded to the outcome: baseline hs-TnT, age, sex, history of cardiovascular disease, heart failure, Charlson Comorbidity Index, metastatic disease, right-sided colorectal cancer, and beta-blocker, ACEI/ARB/ARNI, SGLT2 inhibitor, and VEGF inhibitors such as bevacizumab. MACE lacks a universally accepted definition, and its components vary across studies (22). We therefore prespecified a deliberately broad composite intended to capture all clinically relevant treatment-related cardiovascular side effects and complications that could affect the disease course, including treatment-associated hypertension, a recognized cardiovascular toxicity of fluoropyrimidine- and VEGF-inhibitor-based therapy (12, 13). Baseline hs-TnT was entered as a continuous predictor, first expanded with splines to allow for nonlinear effects and was only used linearly after the non-linearity was deemed insignificant.

A sensitivity analysis was performed to assess the robustness of the findings and to minimize potential confounding from the well-established blood pressure-elevating effect of bevacizumab by excluding treatment-related hypertension from the composite endpoint. Other prespecified sensitivity analyses were planned to account for heterogeneity in MACE mechanisms and clinical presentations, including clustering of events into ischemic, arrhythmic, and vascular categories. However, owing to the low number of events, such subgroup analysis was not feasible, as reliable assessment of the effect of the covariates could not be ensured.

The discriminatory performance of baseline high-sensitivity troponin T (hs-TnT) for predicting MACEs was evaluated using receiver operating characteristic (ROC) curve analysis. The area under the ROC curve (AUC) and its 95% confidence interval were estimated using 2,000 bootstrap replicates. The optimal hs-TnT cut-off value was determined using the Youden index, and the corresponding sensitivity and specificity were calculated.

The incremental prognostic value of hs-TnT beyond conventional clinical predictors was assessed using the adequacy index (25). The fraction of new prognostic information provided by hs-TnT was expressed as one minus the adequacy index.

To further quantify the incremental prognostic contribution of baseline hs-TnT, continuous net reclassification improvement (NRI) was calculated. The reference model included conventional clinical predictors, and the NRI quantified the extent to which adding hs-TnT improved individual patient risk classification.

The potential clinical utility of a model incorporating baseline hs-TnT and clinical predictors was evaluated using decision curve analysis (DCA), which estimates net benefit across a range of threshold probabilities by balancing the benefits of true-positive classifications against the harms of false-positive classifications. Net benefit was compared with the default strategies of treating all patients or treating no patients.

All analyses were conducted in R (version 4.5.0, R Foundation for Statistical Computing, Vienna, Austria) using the rms package for regression modeling and assessment of potential nonlinear associations and the pROC package for ROC curve analyses (26–28).

## 3 Results

### 3.1 Study population

The analytic cohort included 180 patients (median age 69 years, 46.1% female). Metastatic disease was present in 65.6%, and 29.4% received bevacizumab. Baseline comorbidities were common (hypertension 63.3%, diabetes 24.4%, hypercholesterolemia 29.4%), and hs-TnT exceeded the upper reference limit in 18.3% of patients. Detailed baseline characteristics are shown in Table 1.

**Table 1:** Baseline characteristics in the MACE and non-MACE groups.

Variable	MACE (*n* = 44)	Non-MACE (*n* = 136)	All (*n* = 180)	*p*-value
Demographics				
Age (years)	64.5/70.5/78	58/66/75	58/69/75	0.074
Female	20 (45.5)	63 (46.3)	83 (46.1)	0.92
Clinical parameters of colorectal cancer				
Right-sided colorectal cancer	14 (31.8)	32 (23.5)	46 (25.6)	0.27
Metastasis at the diagnosis	32 (72.7)	86 (63.2)	118 (65.6)	0.076
Liver	19 (43.2)	41 (30.1)	60 (33.3)	0.11
Lung	5 (11.4)	8 (5.9)	13 (7.2)	0.22
Previous oncological disease	6 (13.6)	20 (14.7)	26 (14.4)	0.861
Previous chemotherapy	2 (4.5)	11 (8.1)	13 (7.2)	0.43
Chemotherapy				
5-FU-based therapy	44 (100)	136 (100)	180 (100)	0.61
Intravenous	33 (75)	88 (64.7)	121 (67.2)	0.58
Oral (capecitabine)	11 (25)	48 (35.3)	59 (32.8)	0.27
VEGF-inhibitor therapy	23 (52.3)	30 (22.1)	53 (29.4)	<0.001
Laboratory parameters				
Anaemia	17 (38.6)	64 (47.1)	81 (45)	0.33
Baseline troponin value >14 ng/l	20 (45.5)	13 (9.6)	33 (18.3)	<0.001
Medications				
Beta-blocker	18 (40.9)	38 (27.9)	56 (31.1)	0.11
ACEI/ARB/ARNI	28 (63.6)	56 (41.2)	84 (46.7)	0.009
MRA	1 (2.3)	5 (3.7)	6 (3.3)	0.65
SGLT2-inhibitor	1 (2.3)	3 (2.2)	4 (2.2)	0.979
Statin	11 (25)	31 (22.8)	42 (23.3)	0.764
Previous cardiovascular comorbidities and risk factors				
Hypertension	28 (63.6)	86 (63.2)	114 (63.3)	0.962
Diabetes mellitus	9 (20.5)	35 (25.7)	44 (24.4)	0.729
Hypercholesterolemia	14 (31.8)	39 (28.7)	53 (29.4)	0.691
Heart failure	1 (2.3)	14 (10.3)	15 (8.3)	0.094
Acute myocardial infarction	2 (4.5)	4 (2.9)	6 (3.3)	0.606
Atrial fibrillation/flutter	7 (15.9)	18 (13.2)	25 (13.9)	0.656
Coronary disease	4 (9.1)	7 (5.1)	11 (6.1)	0.342
Stent	3 (6.8)	2 (1.5)	5 (2.8)	0.061
Stroke/TIA	1 (2.3)	7 (5.1)	8 (4.4)	0.42
Peripheral artery disease	0 (0)	4 (2.9)	4 (2.2)	0.250
Thromboembolic events	2 (4.5)	7 (5.1)	9 (5)	0.87
Active smoking	8 (18.2)	16 (11.8)	24 (13.3)	0.22
Previous smoking	15 (34.1)	52 (38.2)	67 (37.2)	0.82
Charlson Comorbidity Index	2.75/3/5	2/3/4	2/3/4	0.36
CCI 0–1	10 (22.7)	29 (21.3)	39 (21.6)	0.88
CCI 2–3	16 (36.4)	62 (45.6)	78 (43.3)	0.49
CCI ≥4	22 (50)	45 (33.1)	67 (37.2)	0.19

5-FU: 5-fluorouracil ACEI: angiotensin-converting enzyme inhibitor ARB: angiotensin receptor blocker ARNI: angiotensin receptor-neprilysin inhibitor CCI: Charlson Comorbidity Index MACE: major adverse cardiovascular events MRA: mineralocorticoid receptor antagonist SGLT2: sodium-glucose co-transporter-2 TIA: transient ischemic attack VEGF: vascular endothelial growth factor.

### 3.2 Major adverse cardiovascular events and mortality

During a median follow-up of 185 days (IQR 171–294), 44 of 180 patients (24.4%) experienced 46 MACEs. Complete follow-up was achieved for all enrolled participants. Events occurred predominantly early, with 15 MACEs (32.6%) during the first chemotherapy cycle and 11 (23.9%) during the second. Hypertension was the most frequent component of the composite outcome (20 events, 43.5%), followed by atrial fibrillation/flutter (10 events, 21.7%) and angina/ischemic heart disease (9 events, 19.6%). No acute myocardial infarctions were recorded. Detailed event distribution is provided in Table 2.

**Table 2:** Distribution of major adverse cardiovascular events (MACEs) during follow-up.

Event	Number	%
Number of MACEs	46	100
Patients with two events	2	4.3
MACEs clinical development		
1st cycle of chemotherapy	15	32.6
2nd cycle of chemotherapy	11	23.9
3rd and 4th cycle of chemotherapy	4	8.7
Further cycles of chemotherapy	16	34.8
MACEs		
Hypertension	20	43.5
Atrial fibrillation/flutter	10	21.7
Angina/ischaemic heart disease	9	19.6
Heart failure	2	4.3
Stroke/TIA	1	2.2
Thromboembolic events	4	8.7
Deep vein thrombosis	2	4.3
Pulmonary embolism	1	2.2
Thrombosis of portal vein	1	2.2

MACE: major adverse cardiovascular event; TIA: transient ischemic attack.

Over follow-up, 17 patients (9.4%) died. Only one death was CV, while most resulted from cancer progression or sepsis.

### 3.3 Multivariable and sensitivity analyses

In the initial L2-penalized multivariable logistic regression model, baseline hs-TnT levels (OR 2.35 per 10 ng/L increase, 95% CI 1.37–4.05, *p* = 0.002) and VEGF-inhibitor (bevacizumab) therapy (OR 2.38, 95% CI 1.23–4.60, *p* = 0.010) were independently associated with the occurrence of MACE. In contrast, age, sex, metastatic disease status, prior cardiovascular disease, comorbidity burden as assessed by the Charlson Comorbidity Index, oncological characteristics, and background cardioprotective pharmacotherapy were not significantly associated with MACE after multivariable adjustment. The multivariable model demonstrated good discrimination, with an area under the receiver operating characteristic curve (AUC) of 0.766 (95% CI 0.678–0.855). The results of the multivariable logistic regression analysis are presented in Table 3 and illustrated in Figure 2.

**Table 3:** Predictors of major adverse cardiovascular events (MACEs) in multivariable logistic regression analysis.

Variable	Odds Ratio (OR)	95% confidence interval		*p*-value
		Lower	Upper	
Age (per year)	1.08	0.73	1.60	0.70
Previous cardiovascular disease	1.12	0.47	2.67	0.80
Charlson Comorbidity Index (+1 unit)	0.88	0.69	1.11	0.28
Baseline troponin value (+10 ng/L)	**2** **.** **35**	**1**.**37**	**4**.**05**	**0**.**002**
Male	1.13	0.59	2.16	0.70
Metastasis at the diagnosis	1.04	0.53	2.03	0.92
Right-sided colorectal cancer	1.35	0.69	2.66	0.38
Heart failure	0.53	0.21	1.34	0.18
Beta-blocker therapy	1.26	0.63	2.49	0.51
ACEI/ARB/ARNI therapy	1.64	0.84	3.18	0.15
SGLT2-inhibitor therapy	0.90	0.32	2.53	0.84
VEGF-inhibitor therapy (bevacizumab)	**2**.**38**	**1**.**23**	**4**.**60**	**0**.**010**

ACEI: angiotensin-converting enzyme inhibitor ARB: angiotensin receptor blocker ARNI: angiotensin receptor-neprilysin inhibitor MACE: major adverse cardiovascular event OR: odds ratio SGLT2: sodium-glucose co-transporter-2 inhibitor VEGF: vascular endothelial growth factor.

**Figure 2:**
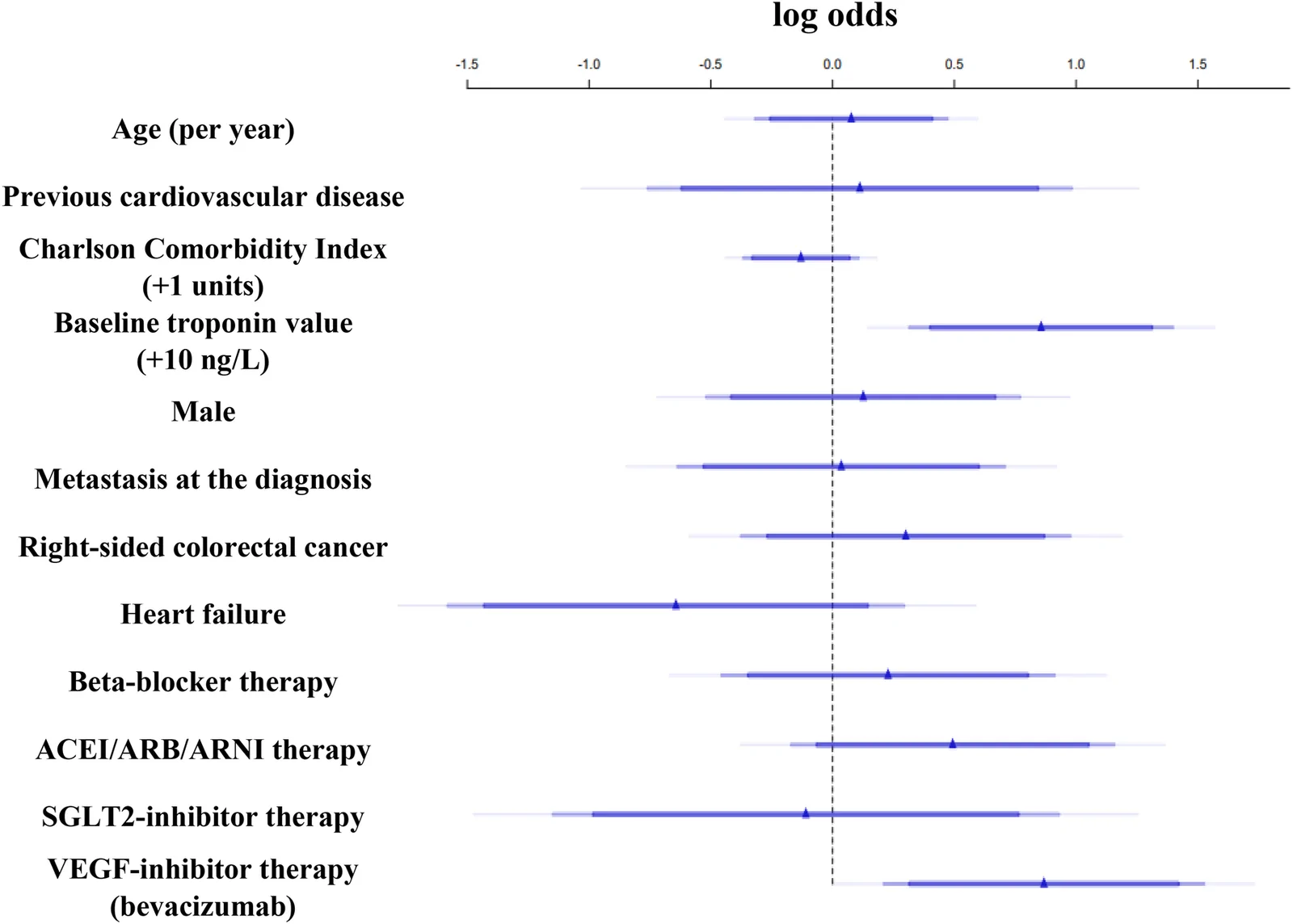
Predictors of major adverse cardiovascular events (MACEs) illustrated on a forest plot. Odds ratios (ORs) with 95% confidence intervals are shown for all variables included in the final model.

To assess the robustness of the findings, a sensitivity analysis was performed excluding treatment-related hypertension from the composite endpoint because of the well-established hypertensive effect of bevacizumab. In this model, baseline hs-TnT remained the sole independent predictor of cardiovascular events, with an effect size comparable to that observed in the primary analysis. For every 10 ng/L increase in baseline hs-TnT, the odds of experiencing an event increased by 65% (OR 1.65, 95% CI 1.03–2.63, *p* = 0.037) (Supplementary Table SM1; Supplementary Figure SM1). In contrast, the previously observed association between VEGF-inhibitor therapy and MACE was attenuated and was no longer statistically significant.

### 3.4 Baseline high-sensitivity troponin T

Both univariable and multivariable analyses demonstrated significant associations between elevated baseline troponin levels and lower eGFR values (univariable: *p* = 0.002, multivariable: OR 0.96, 95% CI 0.94–0.99, *p* = 0.006), as well as older age (univariable: *p* < 0.001, multivariable: OR 1.06, 95% CI 1.01–1.11, *p* = 0.020). In contrast, prior myocardial infarction, hypertension, stroke/TIA, heart failure, and higher Charlson Comorbidity Index values (>6) were not significantly associated with elevated baseline troponin levels in either analysis (Supplementary Tables SM2, SM3).

Receiver operating characteristic (ROC) curve analysis of baseline hs-TnT alone demonstrated modest discrimination for predicting the occurrence of MACE, with an AUC of 0.628 (95% CI 0.513–0.742) (Figure 3). The optimal hs-TnT cut-off value identified using the Youden index was 14 ng/L, corresponding to a sensitivity of 45.5% and a specificity of 90.4% (Figure 4).

**Figure 3:**
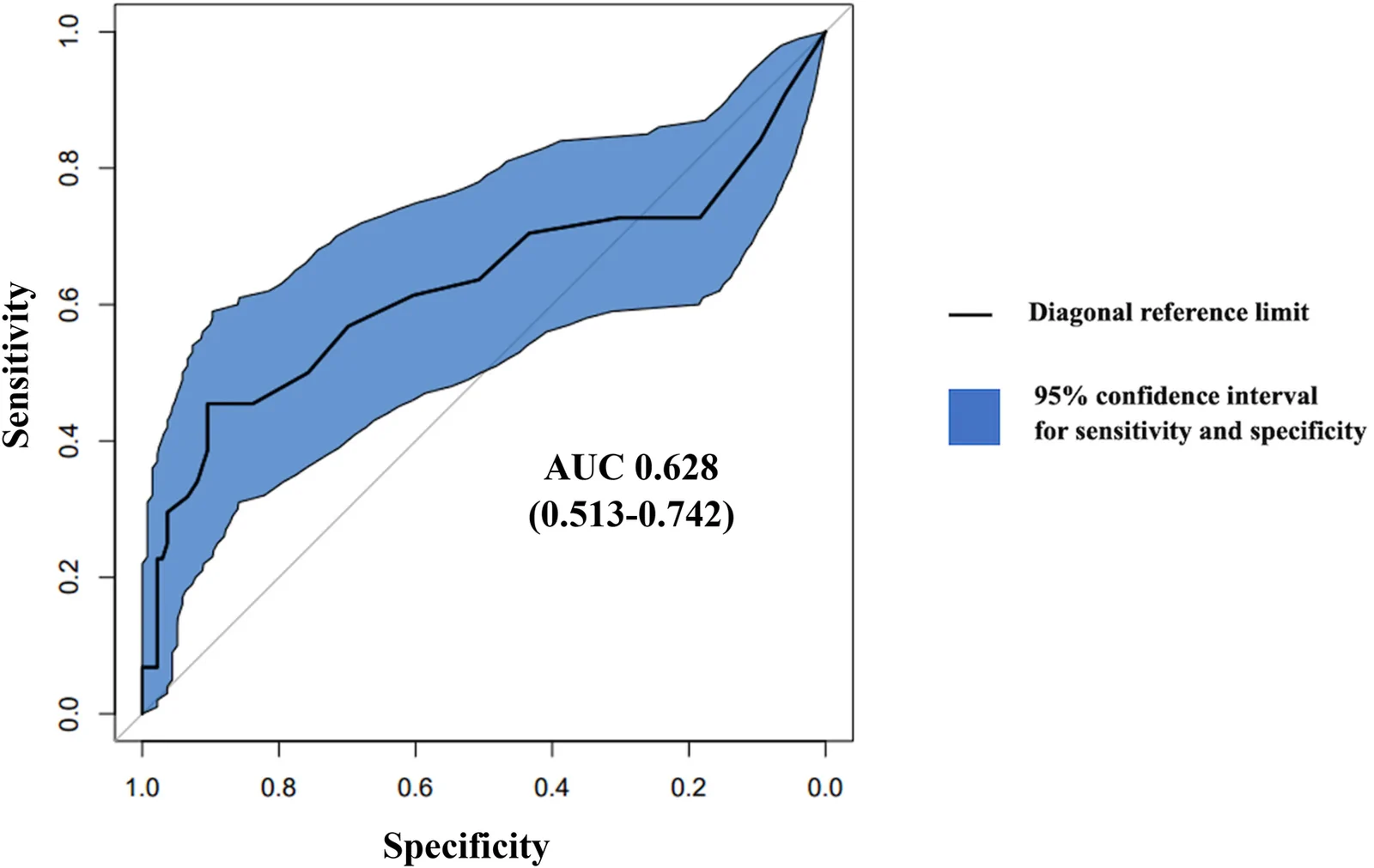
Receiver operating characteristic (ROC) analysis of baseline high-sensitivity troponin T levels for predicting major adverse cardiovascular events (MACEs). The area under the curve (AUC) was 0.628 (95% CI 0.513–0.742). The shaded area represents the 95% confidence interval estimated using 2,000 bootstrap replicates. AUC: area under the curve.

**Figure 4:**
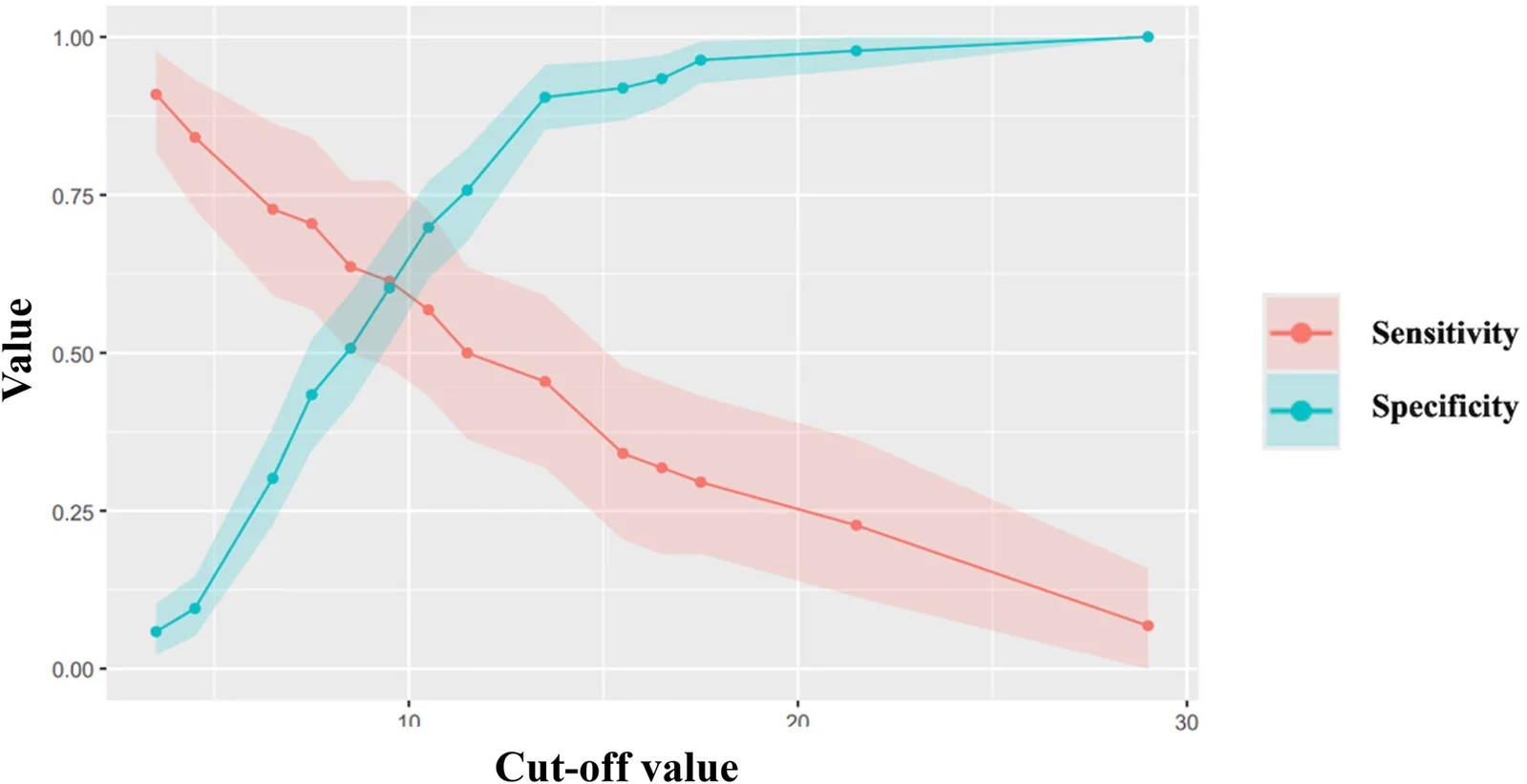
Sensitivity and specificity of baseline high-sensitivity troponin T (hs-TnT) across the range of evaluated cut-off values for predicting major adverse cardiovascular events (MACEs). The optimal cut-off value determined by the Youden index was 14 ng/L, corresponding to a sensitivity of 45.5% and a specificity of 90.4%. The shaded areas surrounding the sensitivity and specificity curves represent the estimated 95% bootstrap confidence intervals.

Adequacy index analysis demonstrated that baseline hs-TnT contributed 38.3% of the total prognostic information provided by a multivariable model. Furthermore, inclusion of baseline hs-TnT significantly improved model performance compared with the model including clinical predictors alone (*p* < 0.001), supporting its incremental prognostic value.

### 3.5 Net reclassification improvement (NRI) and decision curve analysis (DCA)

Continuous net reclassification improvement (NRI) analysis demonstrated positive reclassification across most threshold probabilities, indicating improved individual patient risk classification following the addition of baseline hs-TnT to the clinical prediction model. The greatest improvement was observed at intermediate threshold probabilities, whereas little additional benefit was evident at very high threshold probabilities (Supplementary Figure SM2).

Decision curve analysis (DCA) demonstrated greater net clinical benefit of the final multivariable prediction model than the default strategies of treating all patients or treating none across a broad range of clinically relevant threshold probabilities, supporting its potential clinical utility (Supplementary Figure SM3).

## 4 Discussion

In this prospective cohort of patients with CRC receiving fluoropyrimidine-based chemotherapy, baseline hs-TnT was an independent predictor of treatment-associated MACEs. In the primary multivariable analysis, baseline hs-TnT and VEGF-inhibitor therapy were the only independent predictors of MACE, whereas hs-TnT remained the sole independent predictor after exclusion of treatment-related hypertension in the sensitivity analysis. Most cardiovascular events occurred during the first two chemotherapy cycles, suggesting that baseline cardiovascular vulnerability becomes clinically relevant early after treatment initiation.

Elevated baseline hs-TnT concentrations were independently associated with lower eGFR and older age, consistent with previous reports demonstrating that impaired renal function, age-related myocardial remodeling, and chronic subclinical myocardial injury contribute to persistently elevated troponin concentrations (29, 30). These findings suggest that renal dysfunction and age-related cardiovascular changes may exert a greater influence on baseline hs-TnT concentrations than the investigated comorbid conditions, several of which may primarily reflect overall patient frailty rather than active myocardial injury.

More than half of all cardiovascular events occurred during the first two chemotherapy cycles, indicating that toxicity developed predominantly during the early phase of treatment. This temporal pattern is consistent with previous reports of fluoropyrimidine-associated cardiotoxicity, in which ischemic symptoms and hemodynamic instability typically develop shortly after treatment initiation rather than during prolonged exposure (7, 8). Our findings extend these observations by demonstrating that this early-risk period encompasses a broader spectrum of cardiovascular complications, supporting intensified cardiovascular surveillance during the initial chemotherapy cycles rather than uniform monitoring throughout treatment.

The present study was conducted in a population characterized by an exceptionally high burden of both cardiovascular disease and CRC, a context that remains underrepresented in the existing literature. In Hungary, cardiovascular disease accounts for more than half of all deaths, while CRC mortality is the highest in the European Union and survival outcomes remain among the poorest in Europe (31–33). This dual high-risk epidemiological setting likely contributed to the substantial comorbidity burden observed in our cohort, reflected by the high proportion of patients with Charlson Comorbidity Index scores ≥4 (37.2%), exceeding those reported in CRC cohorts from other regions (34–36).

Against this background, our findings emphasize the importance of interpreting cardiovascular risk within its epidemiological context. Although the distinctive characteristics of the study population may limit direct generalizability, they also represent an important strength by enabling evaluation of cardiovascular risk stratification in a particularly vulnerable patient population. External validation in cohorts with different cardiovascular risk profiles will be essential to determine the broader applicability of these findings.

Baseline hs-TnT demonstrated independent prognostic value before chemotherapy initiation. Similar associations between pre-treatment hs-TnT elevation and subsequent cardiovascular complications have been reported in patients receiving anthracyclines and HER2-targeted therapies (16–18), whereas prospective evidence in fluoropyrimidine-treated CRC has remained limited. Our study extends these observations by providing prospective CRC-specific evidence supporting baseline hs-TnT as a marker of underlying cardiovascular vulnerability that becomes clinically relevant following initiation of systemic therapy.

Although baseline hs-TnT and bevacizumab therapy were independently associated with cardiovascular events in the primary multivariable analysis, exclusion of treatment-related hypertension identified hs-TnT as the sole independent predictor, indicating that its prognostic value was not solely explained by therapy-induced blood pressure elevation. Receiver operating characteristic analysis demonstrated only modest discrimination of baseline hs-TnT as a standalone biomarker (AUC 0.628, 95% CI 0.513–0.742), which is not unexpected given the multifactorial pathophysiology of treatment-associated cardiovascular events. Rather than serving as an isolated prediction tool, hs-TnT appears to provide complementary prognostic information when interpreted together with established clinical predictors and treatment characteristics. This is biologically plausible, as baseline hs-TnT may reflect underlying myocardial vulnerability, whereas VEGF-inhibitor therapy primarily contributes treatment-related vascular stress through distinct pathophysiological mechanisms (37, 38). These findings are further supported by the adequacy index analysis, which demonstrated that baseline hs-TnT accounted for 38.3% of the prognostic information provided by the final prediction model. In addition, positive continuous net reclassification improvement and favorable decision curve analysis indicated improved patient risk classification and greater potential clinical utility after incorporation of hs-TnT into the prediction model. As the aim of the present study was not to develop or validate a clinical prediction model, formal internal validation and calibration assessment were beyond its scope and should be addressed in future studies specifically designed for clinical prediction model development. Nevertheless, the present prospective findings provide an important foundation for future prospective multicenter studies and clinical investigations aimed at further validating the prognostic and clinical utility of baseline hs-TnT in this setting.

Taken together, these findings support a pragmatic, biomarker-informed approach to cardiovascular risk stratification in patients receiving fluoropyrimidine-based chemotherapy. Rather than functioning as a standalone prediction tool, baseline hs-TnT may complement conventional clinical assessment by identifying patients who could benefit from closer cardiovascular follow-up and early cardio-oncology involvement during the initial chemotherapy cycles. Although the present findings do not support routine universal cardiovascular screening based on hs-TnT alone, they provide prospective CRC-specific evidence supporting its incorporation into structured baseline cardiovascular risk assessment.

An important consideration in the interpretation of our findings is the broad and heterogeneous nature of the composite endpoint. The endpoint was prospectively defined to comprehensively capture the spectrum of clinically relevant complications occurring during fluoropyrimidine- and VEGF inhibitor-based anticancer therapy. Accordingly, in addition to ischaemic, arrhythmic, and heart failure events, the composite endpoint also included treatment-related hypertension and venous thromboembolic events.

Although the definition and composition of MACE vary across studies and no universally accepted definition exists, the composite endpoint used in our study reflects a broader spectrum of cardiovascular outcomes. Importantly, each of its components has previously been considered a relevant cardiovascular complication in the existing literature, and none represents a novel or unprecedented component in this context. Nevertheless, the individual components may differ substantially with respect to their underlying pathophysiology and clinical implications.

While the present findings provide clinically relevant insights, they should be interpreted in light of the study's limitations. This was a single-center study conducted in a tertiary referral setting with a selected cohort of patients undergoing standardized longitudinal follow-up, and the sample size and number of outcome events were modest, therefore, an L2-penalized regression framework was applied to reduce the risk of model overfitting. In addition, follow-up was designed primarily to capture early treatment-associated cardiovascular events, and later complications may therefore be underrepresented. Consequently, the limited number of events precluded reliable prespecified subgroup analyses according to MACE subtype and may have influenced the precision of the estimated associations. A further limitation of our study is the potential for ascertainment bias, as elevated hs-TnT levels may have prompted closer clinical attention and, when clinically indicated, additional cardiological evaluation. However, hs-TnT levels were measured in all patients before each chemotherapy cycle, and elevated troponin values were not interpreted as isolated laboratory abnormalities but were assessed in an integrated manner alongside the patient's overall clinical condition and any cardiovascular symptoms or clinical manifestations. Cardiovascular events were adjudicated according to predefined clinical criteria, which may have mitigated the potential impact of this bias. Despite these limitations, the present findings provide a foundation for future research, supporting the need for multicenter studies to confirm the results and further evaluate the clinical utility of biomarker-guided cardiovascular surveillance strategies.

## 5 Conclusions

In this prospective cohort of patients with CRC receiving fluoropyrimidine-based chemotherapy, baseline hs-TnT was independently associated with treatment-related MACEs occurring predominantly during the early phase of therapy. Although its discriminatory performance as a standalone biomarker was modest, hs-TnT provided incremental prognostic information beyond conventional clinical predictors and may complement baseline cardiovascular risk assessment by identifying patients who could benefit from intensified cardiovascular surveillance during the initial chemotherapy cycles. These findings support further evaluation of biomarker-guided cardiovascular risk stratification in prospective multicenter studies before its implementation in routine clinical practice.

## Data Availability

The raw data supporting the conclusions of this article will be made available by the authors, without undue reservation.
