# Supplementary material for: High-sensitivity troponin T as a predictor of cardiovascular events in colorectal cancer patients undergoing chemotherapy: results of the COLOVASC prospective cohort study
Source: Front Cardiovasc Med. 2026 Oct 13;13:1943142. doi: 10.3389/fcvm.2026.1943142 (PMC13710027; doi:10.3389/fcvm.2026.1943142)
Supplement: Supplementary file 1 [file Datasheet1.pdf]

Supplementary Material

Table SM1. Predictors of major adverse cardiovascular events (MACEs) after exclusion of treatment-related hypertension

	Odds Ratio (OR)	95% confidence interval		p-value
**Age (per year)**	1.08	0.80	1.45	0.607
**Previous cardiovascular disease**	1.07	0.51	2.27	0.852
**Charlson Comorbidity Index +1 units**	0.96	0.81	1.12	0.580
**Baseline troponin value +10 ng/L**	**1.65**	**1.03**	**2.63**	**0.037**
**Male**	1.11	0.67	1.83	0.684
**Metastasis at the diagnosis**	1.12	0.67	1.86	0.671
**Right-sided colorectal cancer**	1.27	0.76	2.12	0.362
**Heart failure**	0.81	0.45	1.46	0.487
**Beta-blocker therapy**	1.09	0.65	1.84	0.733
**ACEI/ARB/ARNI therapy**	1.36	0.82	2.26	0.230
**SGLT2-inhibitor therapy**	1.00	0.54	1.85	0.995
**VEGF-inhibitor therapy (bevacizumab)**	1.21	0.72	2.03	0.483

Table SM2. Univariable analysis of clinical factors associated with elevated baseline troponin levels

Variable	p-value
**eGFR**	**0.002**
**Age**	**<0.001**
**Acute myocardial infarction**	1
**Hypertension**	0.117
**Stroke/TIA**	0.361
**Heart failure**	0.251
**Higher Charlson Comorbidity Index values (>6)**	0.161

eGFR: estimated glomerular filtration rate, TIA: Transient Ischemic Attack

Table SM3. Multivariable analysis of clinical factors associated with elevated baseline troponin levels

Variable	Odds Ratio	95% CI	p-value
**eGFR**	**0.96**	**0.94-0.99**	**0.006**
**Age**	**1.06**	**1.01-1.11**	**0.020**
**Acute myocardial infarction**	0.21	0.01-4.06	0.301
**Hypertension**	1.35	0.51-3.59	0.549
**Stroke/TIA**	2.89	0.56-15.08	0.207
**Heart failure**	2.27	0.51-10.03	0.281
**Higher Charlson Comorbidity Index values (>6)**	0.71	0.21-2.37	0.572

eGFR: estimated glomerular filtration rate, TIA: Transient Ischemic Attack

Figure SM1. Predictors of major adverse cardiovascular events (MACEs) after exclusion of treatment-related hypertension illustrated on a forest plot

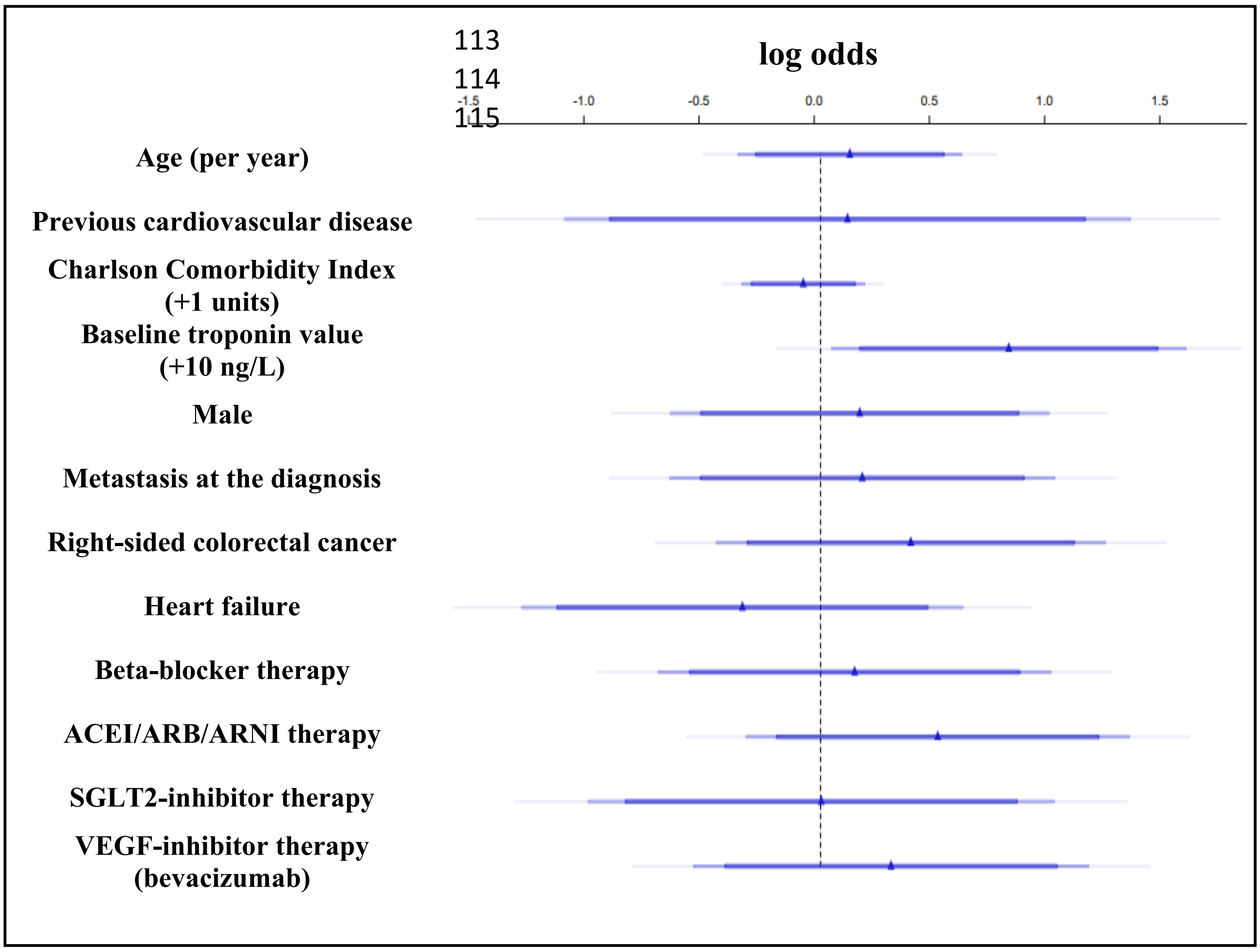

Figure SM2. Continuous net reclassification improvement (NRI) across threshold probabilities

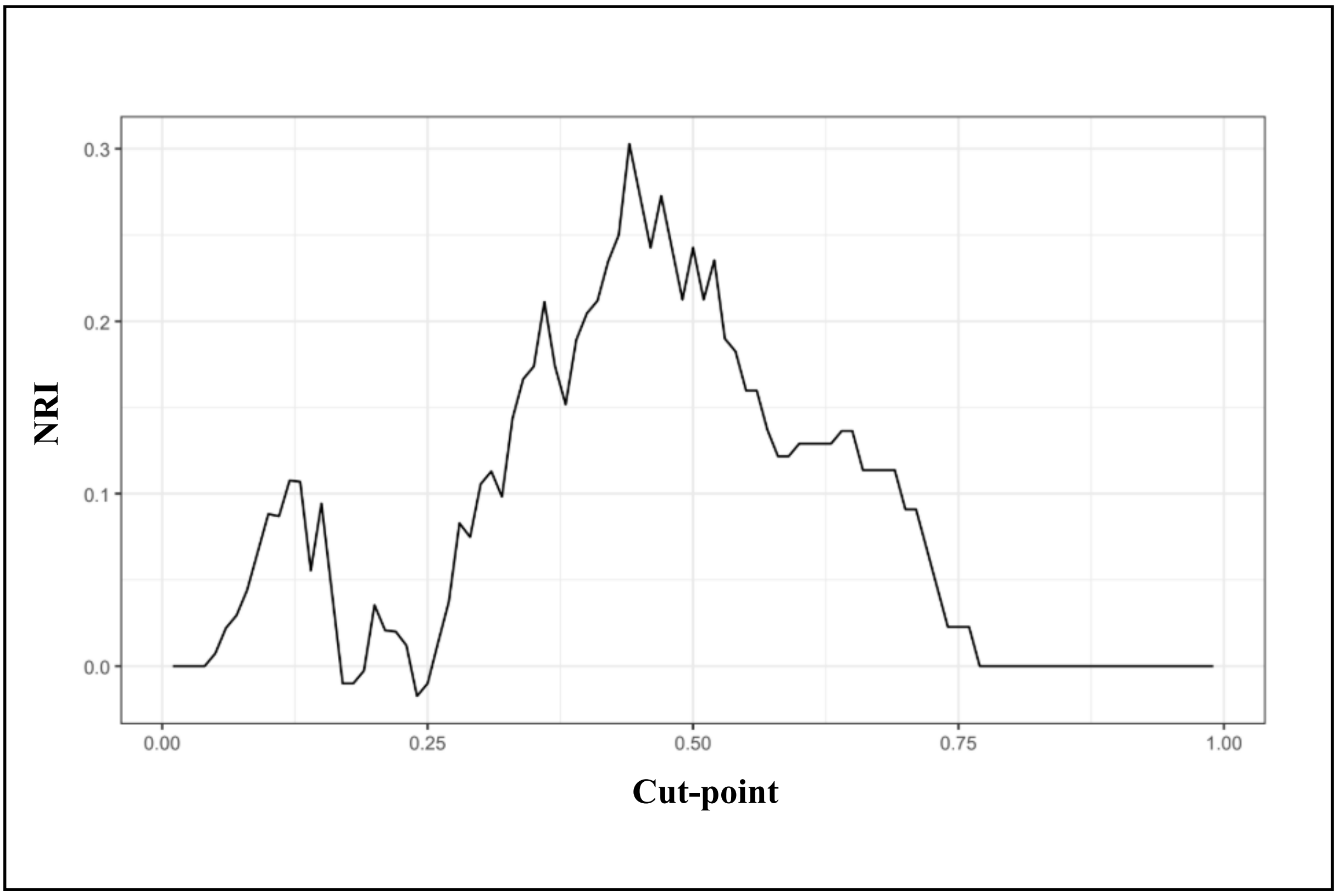

Positive NRI values indicate improved individual patient risk classification after the addition of baseline hs-TnT to the clinical prediction model compared with the reference model containing clinical predictors alone.

Figure SM3. Decision curve analysis (DCA)

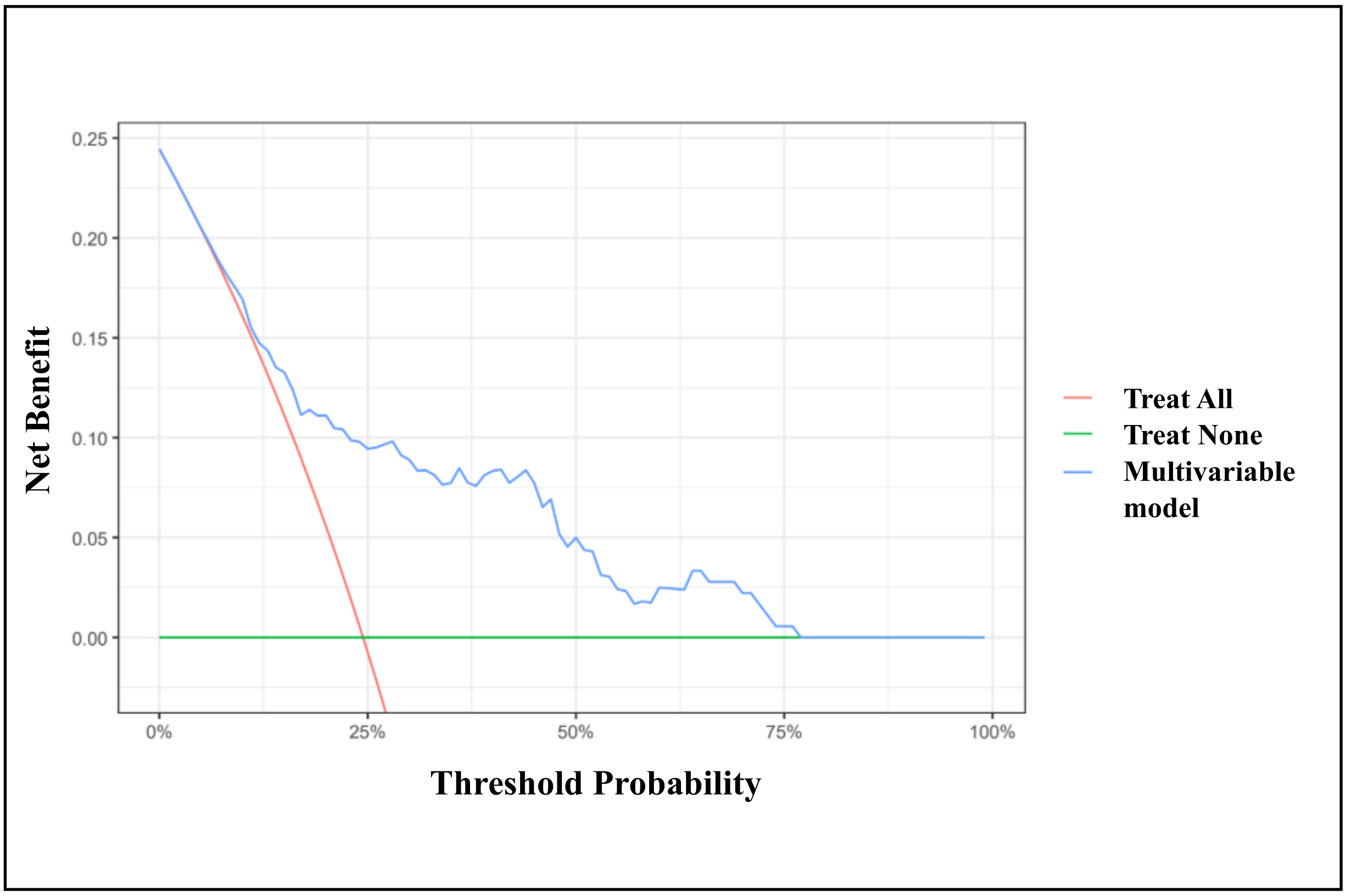

The blue curve represents the prediction model, whereas the red and green curves represent the default strategies of treating all patients and treating no patients, respectively. Across a broad range of clinically relevant threshold probabilities, the prediction model provided greater net benefit than either default strategy.
